# The Host Coral Bleaching Response Viewed Through the Lens of Multi‐Omics: Multi‐Omics Provides the Tools to Understand the Complex Molecular Basis of Coral Bleaching, Which Can Aid Conservation Efforts

**DOI:** 10.1002/bies.70110

**Published:** 2026-01-26

**Authors:** Debashish Bhattacharya, Shrinivas Nandi, Erin E. Chille, Miriam Arroyo, Timothy G. Stephens

**Affiliations:** ^1^ Department of Biochemistry and Microbiology Rutgers University New Brunswick New Jersey USA; ^2^ Microbial Biology Graduate Program Rutgers University New Brunswick New Jersey USA; ^3^ Ecology and Evolution Graduate Program Rutgers University New Brunswick New Jersey USA

**Keywords:** coral bleaching, coral reefs, dysbiosis, holobiont, multi‐omics, ocean warming, reactive oxygen species, redox stress

## Abstract

We review recent multi‐omics analyses of the coral heat stress response to explore the generality of the Oxidative Theory of Coral Bleaching (OTCB), which posits that algal symbiont release is the final act of defense by the coral host to survive alga‐derived oxidative stress. The OTCB is particularly relevant given that ocean warming, which is accelerating under climate change, has proven devastating for corals, leading to the bleaching phenotype and widespread reef loss. Multi‐omics results, in combination with other data, such as genome‐wide association studies, support the idea that coral bleaching is a multifactorial response that reflects a wide array of causes and effects and is population‐specific under most conditions, with coral ploidy and genotype being critical to bleaching sensitivity. This perspective leverages the location, algal and prokaryotic microbiome, and host genotype‐specific aspects of coral resilience to promote a new “personal genomics” approach to coral conservation, analogous to that used in human health.

## Graphical abstract

Coral bleaching is driven by multiple inputs, with heat stress and/or high irradiance being most important. The bleaching response is multifactorial with host animal species/strain and algal symbiont genotypes being critical features. Omics readout of heat stress responses includes gene expression, proteomics, metabolite, and SNP data with transcript changes most impacted by genotype and local adaptation.

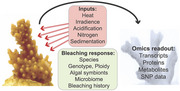

**Abbreviations**
DEG: differentially expressed gene
GDE: genotype‐dependent expression
OTCB: Oxidative Theory of Coral Bleaching
RNS: reactive nitrogen species
ROS: reactive oxygen species
TDE: treatment‐driven expression

## 1 Introduction

Coral “**bleaching**” (see Box), which results in a ghostly white appearance of colonies, is caused by the expulsion of algal symbionts (Symbiodiniaceae dinoflagellates) by the host animal or the loss of algal pigments. Symbiodiniaceae provide energy via photosynthetically fixed carbon and other nutrients to sustain the coral holobiont [1, 2, 3, 4]—the genetically diverse collection of organisms (cnidarian animal host, Symbiodiniaceae, prokaryotes, fungi, protists, and viruses) that comprise the coral **holobiont** (see Box) and contribute to its health and resilience [5, 6, 7, 8]. Within the coral host, Symbiodiniaceae are housed in specialized compartments referred to as **symbiosomes** (see Box), which are supplied with inorganic carbon and acidified to improve algal photosynthetic efficiency [9]. The cnidarian hosts also regulate the transfer of ammonium and/or amino acids to the algae to control these populations and maintain a stable symbiosis [10]. Heat stress (herein, “thermal stress”) is generally recognized as the leading cause of coral stress and mortality worldwide, with higher ocean temperatures resulting in holobiont **dysbiosis** (see Box) [11], which can lead to bleaching [12, 13, 14]. Heat stress‐driven bleaching can occur when the water temperature is even moderately elevated (e.g., 1–2°C) above the average summer maximum [15]. Other factors may also induce bleaching, including cold, hypoxia, nitrogen enrichment, ocean acidification, and suspended sediments [16, 17]. Several explanations for bleaching have been put forth, the most highly recognized of which is the *Oxidative Theory of Coral Bleaching* (OTCB) (see Box) [18, 19], which proposes that reactive oxygen species (ROS) produced by the endosymbiont is the primary stressor driving the cascade leading to the bleaching phenotype [20]. Given its importance to coral reef loss [21], much effort has focused on validating the existing explanations for coral bleaching and understanding the factors that contribute to bleaching resistance and resilience vis‐à‐vis the holobiont.

**BOX**

- **Antioxidant**: Any substance that significantly slows or prevents the oxidation of another molecule by neutralizing chemically reactive species. The latter includes free radicals or other reactive oxygen/nitrogen species (ROS/RNS), which need to be neutralized before they can damage lipids, proteins, nucleic acids, or other cellular components.
- **Coral bleaching**: A stress response in which reef‐building corals (Scleractinia) expel most of the dinoflagellate algal symbionts (Symbiodiniaceae) that live inside their tissues and provide most of the metabolic energy to the cnidarian host via photosynthesis. Loss of algal symbionts confers a ghostly white (“bleached”) appearance to coral colonies that reveals the underlying calcium‐carbonate skeleton.
- **Coral dysbiosis**: Disruption or imbalance in the composition, abundance, or functioning of the coral holobiont microbial community that can lead to bleaching, increased disease risk, or other forms of damage to the colony.
- **Coral holobiont**: The community of organisms comprising the cnidarian coral host and associated microbiome, that includes Symbiodiniaceae (algal symbionts), bacterial and archaeal cells, viruses, fungi, and other microorganisms.
- **Oxidative Theory of Coral Bleaching (OTCB)**: This theory posits that algal symbiont release by the coral host is a response to extreme oxidative stress produced by photosynthetic algal symbionts, often due to ocean warming events.
- **Reactive oxygen species (ROS)**: These are oxygen‐derived molecules that are more chemically reactive than ground‐state O_2_ and include O_2_^−^ (superoxide anion), H_2_O_2_ (hydrogen peroxide), OH (hydroxyl radical), and ^1^O_2_ (singlet oxygen). ROS are a by‐product of aerobic life and produced in cells by the mitochondrial electron‐transport chain, and in photosynthetic cells, also by Photosystems I and II. ROS are important signaling molecules, but in excess, lead to oxidative stress.
- **Symbiosome**: Specialized, host‐derived membrane compartments that enclose individual Symbiodiniaceae algae in the coral gastrodermal cell layer. Symbiosomes integrate host and algal metabolism, mediate immune tolerance, and respond to environmental changes, such as thermal stress.

## 2 The Complex Nature of the Coral Thermal Stress Response

The multi‐factorial nature of the coral holobiont response to thermal stress is becoming increasingly recognized. Unlike diseases that may have a single infectious agent, bleaching reflects the sum of all interactions between holobiont members and the environment [22]. If, as has recently been demonstrated, coral host ploidy and genotype play important roles in resilience [23, 24, 25, 26], then it will likely prove very challenging to disentangle the contributions of host genotype, algal genotype (these algae are highly complex with nuclear genomes ca. 1 Gbp in size with >30K genes) [27], and other members of the holobiont that vary not only within species but also among different species that may have different evolutionary trajectories vis‐à‐vis reproduction, population size, polyploidy, among other parameters [23]. For these reasons, the term *coral*, which describes an ecological lifestyle and shared set of biomineralizing morphologies (i.e., in the Scleractinia [stony corals]), that originated in the Triassic over 200 million years ago [28], does not adequately convey the massive genetic diversity present in this lineage. This diversity makes it highly challenging to find unified solutions to the phenotypically complex bleaching behavior. Symbiont switching, the gradual or rapid (i.e., after bleaching) change in composition of algal symbiont genotypes, often in response to environmental factors, adds additional layers of complexity to the holobiont [29]. The prokaryotic microbiome can be just as dynamic and is susceptible to destabilization upon exposure to perturbations, such as environmental change or the application of broad‐scale antibiotics [30, 31, 32]. Although algal symbiont dynamics and the prokaryotic microbiome clearly play important roles in enhancing coral resistance and resilience to thermal stress [33, 34], in this study, as described in Section 1, we focus on the coral host, using the OTCB [18, 19] paradigm to explore novel ways to think about this problem [35].

## 3 Nature of the Holobiont and Study Limitations

Before delving more deeply into the OTCB, we describe the approach taken in this paper. There are differing views about how to interpret biotic interactions within holobionts writ large. One perspective is of holobionts as biological individuals underpinned by mutualistic associations to ensure common benefit, which comprise the unit of selection, that is, the hologenome theory of evolution [36]. A differing view is of holobionts as ecological communities. From the host perspective, they act as biological individuals; however, from the microbe perspective, they are communities of independent individuals, that is, part‐dependent [37]. The asymmetry in the latter view means holobionts fulfill conditions for being physiological individuals when approached from the host perspective, but do not qualify as biological individuals from the microbe perspective (i.e., they are communities). The latter view stresses biotic interactions that under mutualism can drive holobiont evolution [38]. From another perspective, the stable microbiome was described by Tripp et al. as the symbiome, which includes colocalized and coevolving taxa (i.e., under selection) comprising a given consortium, excluding sporadic associations [39]. We consider all these assessments to be useful under different circumstances. For example, thinking of coral colonies as the sum of their physiology or metabolism can help us understand more broadly how biological individuals respond to local conditions, which may prove useful for reef conservation. However, the individual roles played by microbes, such as coral algal symbionts, are unassailable when considering coral bleaching or disease. Such is the case with stony coral tissue loss disease (SCTLD), which is likely caused by virus‐driven microbiome dysbiosis [40, 41, 42]. For the algae, in previous work, we proposed the “stepping‐stone model” to explain the evolution of Symbiodiniacae, most of which are facultative lineages with both free‐living and symbiotic life‐history stages. This theory posits that algal symbiont biodiversity is enhanced under the coral symbiosis that selects fast‐growing “winners” in the (under ambient conditions) sheltered, nutrient‐rich holobiont environment. These dominant algal genotypes grow to high numbers in coral symbiosomes and seed the environment once released, due to normal turnover or stress‐related bleaching [33]. Different algae provide different host benefits; therefore, the composition of the algal microbiome is clearly of importance and changeable [27]. Hence, the parts‐list for coral holobionts is rich and complex, and biotic interactions vary based on multiple factors such as local stress factors, host and algal genotype, prokaryotic microbiome composition, and associated viruses.

The decision to focus here on coral host data *in hospite* (i.e., in the coral and not in isolated cultures) was made for the following two reasons. First, the symbiotic state markedly impacts transcriptional responses. Bellantuono et al. [43] found in the coral algal symbiont, *Durusdinium trenchii*, that under heat stress, most differentially expressed genes (DEGs), when comparing *in hospite* versus free‐living cells, were downregulated indicating that the symbiotic state is associated with the shutdown of numerous processes required for the free‐living lifestyle. Specifically, free‐living cells showed upregulation of genes involved in cell signaling pathways, environmental responses, and genes related to mitosis, meiosis, and motility, whereas *in hospite*, algal cells had higher expression of genes involved in photosynthesis and carbohydrate transport. Second, given this constraint, it is noteworthy that the current state of coral holobiont multi‐omics delivers host‐dominated data. Whereas deep metagenomics can reconstruct host and microbiome genome sequences in many instances [44] and will improve in future iterations of genomic methods (e.g., Aquatic Symbiosis Genomics Project, Sanger Institute), our work shows that for colonies of the coral *Montipora* spp. collected from Ulithi Atoll in Yap State (Federated States of Micronesia), the program Kraken2 classified <1% of DNA reads derived from colony fragments (i.e., polyps and mucus) to be of bacterial origin and only ∼1%–3% were attributed to Symbiodiniaceae, with the overwhelming majority (>97%) assigned to the coral host reference genome (unpublished data, Bhattacharya lab). In a study of metagenomes from SCTLD‐infected corals from the Dominican Republic, ∼87% of the DNA reads mapped to the coral host *Diploria labyrinthiformis* with the remainder (highly useful data) mapping to the microbiome, including algal symbionts, bacteria, and viruses [42]. These studies underscore how challenging it can be to fully characterize the non‐host fraction of coral holobionts when using standard metagenomic methods. Even if the list of metagenome‐assembled genomes (MAGs) from coral holobionts is relatively complete, the activity of the parts‐list is even more difficult to address. This is because, in the case of widely used poly(A) enriched RNA‐seq data that captures the host coral and algal symbiont (i.e., eukaryotic), only about 10% of the reads map to (often comprising mixed populations) algal genomes, making it difficult to assess their stress responses. This issue is exacerbated by the fact that most dinoflagellate genes are constitutively expressed, regardless of growth condition, with regulation occurring post‐transcriptionally [45, 46]. Proteomics, which is highly informative of holobiont stress responses (see Section 4), is also strongly skewed toward host data with minimal algal and prokaryote microbiome contribution. A recent study showed that for three Australian coral species, only about 11%–13% of the identified host coral and Symbiodiniaceae proteins were alga derived [47]. Finally, interpretation of holobiont metabolomics data is hampered by the vast number of “dark” (unknown) metabolites present in these datasets and the absence of “address labels” for known features [48]. Given these considerations, while keeping in mind the important contributions of diverse holobiont members, we explored the OTCB primarily from the host perspective, which comprises the lion's share of omics data available for interpretation.

## 4 The OTCB and Evidence for and Against Its Generality

The OTCB emphasizes the view that coral bleaching is primarily a cellular response to algal oxidative imbalance [19], which harms the coral host, but may or may not have negative impacts on algal symbiont (see above) and prokaryotic fitness [49, 50]. Lesser [20] proposed that irradiance is critical to understand coral bleaching, with thermal stress experiments done at lower irradiances (e.g., photosynthetically active radiation [PAR] < 200 µmol quanta m^−2^ s^−1^) producing a host‐driven response dominated by reactive nitrogen species (RNS, such as nitric oxide [NO]), whereas under higher irradiances (e.g., PAR > 500 µmol quanta m^−2^ s^−1^), ROS produced by algal symbionts, due to hyperoxia, is the primary stressor that precipitates cell death in the holobiont. The combined impacts of warming waters and high incident light levels are most severe, with the equilibrium of ROS production and antioxidant defense mechanisms in the coral host and algal symbionts being disrupted. That is, under ambient conditions, ROS, such as superoxide radicals (O_2_^−^), hydrogen peroxide (H_2_O_2_), and hydroxyl radicals (OH), are continuously produced at low levels as byproducts of photosynthesis and mitochondrial respiration. The coral holobiont mitigates the oxidative damage resulting from ROS through efficient antioxidant mechanisms, including enzymatic (e.g., superoxide dismutase, catalase) and non‐enzymatic antioxidants (e.g., glutathione, carotenoids). However, high levels of stress‐induced increases in ROS may overwhelm these defenses, causing damage to lipids, proteins, and DNA, ultimately triggering coral bleaching via mechanisms such as expulsion or degradation of Symbiodiniaceae, or their pigments (for an excellent review, see [16]).

Several lines of evidence support the occurrence of oxidative stress in the coral host during high‐temperature challenges in the coral host. These analyses utilized oxidative stress biomarkers (e.g., lipid peroxidation products, oxidized proteins) to demonstrate increases in expression for corals exposed to bleaching conditions, preceding algal symbiont loss [19, 35, 51]. In addition, there was increased expression and activity of native antioxidant enzymes in coral tissues experiencing environmental insults, which suggests that corals actively respond to oxidative stress, supporting the role of ROS in bleaching [22]. Moreover, the application of exogenous antioxidants (e.g., ascorbate + catalase, catechin, and mannitol) in lab studies of the sea anemone model *Exaiptasia diaphana* partially mitigated bleaching responses, which again suggested a causal relationship between oxidative stress and coral bleaching [52]. Additional proof for the link between oxidative stress in the host and thermal bleaching comes from several lines of evidence linking bleaching resistance with higher antioxidant capacity. An example of this is a comparative study of the corals *Acropora millepora* and *Montipora digitata*, which underlined the divergent responses of different coral species to thermal stress, with the photophysiology of *A. millepora* being more impacted by the treatment than *M. digitata* [22]. In a similar study, differences in bleaching severity and bleaching‐related mortality between *Montipora capitata* and *Pocillopora acuta* were attributed to baseline differences in host antioxidant capacity with lower antioxidant capacity contributing to higher sensitivity in *P. acuta* [53]. Differences in bleaching tolerance due to differences in antioxidant capacity have also been found within the same population. In an analysis of thermally sensitive and resilient members of *Acropora hyacinthus* populations, the resilient group constitutively expressed 60 genes, which were overrepresented for antioxidant enzymes and may comprise a “frontloaded” set that presumably are constitutively upregulated to reduce bleaching risk in the more stressful habitat in which these colonies had been drawn from [54]. Taken together, these results implicate the contribution of oxidative stress to bleaching in the coral host. However, which members of the holobiont are contributing to, and in which amount, to the increasing pool of ROS are less well understood.

Although oxidative stress clearly plays a role in the host response to stress, there is conflicting evidence that alga‐derived ROS drives coral bleaching. Analysis of algal symbionts reveals that thermal and high light stress (as expected) do impair photosynthetic efficiency and result in increased ROS production [55]. This well understood link between a compromised photosynthetic machinery and increased oxidative stress in the plastid‐encoded electron transport chain (e.g., the Photosystem II water oxidation reaction) has been demonstrated in many photosynthetic organisms [56, 57] and is consistent with the OTCB [58]. However, several studies have also presented evidence that is inconsistent with the symbiont‐derived ROS hypothesis. For example, in the same *E. diaphana* study described above, in which the application of exogenous antioxidants partially mitigated bleaching, the authors did not find a significant change in net ROS for isolated Symbiodiniaceae cells in their experiments, suggesting that bleaching may occur independently of ROS escape from algal symbionts [59]. Mitochondrial dysfunction is more likely to contribute to algal expulsion, whereby ROS leakage from mitochondria triggers programmed cell death in host cells, amplifying bleaching effects and suggesting host‐derived ROS plays a larger role in the bleaching response [60] (see discussion of multi‐omics data below). Another study, which used microsensors at the tissue interface of the coral *Pocillopora damicornis* to measure hydrogen peroxide and oxygen levels, found no evidence that algal produced H_2_O_2_ was the trigger of coral bleaching under heat stress [61]. In a similar study, single‐cell analysis of fluorescent dyes that allow semi‐quantitative measurement of general ROS in the coral *P. damicornis* exposed to thermal stress showed no evidence that coral algal symbionts were compromised by the heat treatment. These authors concluded that in the absence of severe Photosystem II damage that would precipitate ROS leakage from algae to the host, the symbionts likely did not trigger the bleaching response [59]. Finally, in the comparative study of *A. millepora* and *M. digitata* described above, Krueger et al. [22] found that whereas the coral hosts responded to higher temperatures via the upregulation of catalase, the algal symbiont antioxidant defenses did not show the same trend. All these results support the decoupling of host‐algal thermal stress responses in bleaching, with the host having sufficient scavenging activity to deal with alga‐produced hydrogen peroxide.

Beyond redox‐based responses, thermal stress can also lead to other negative outcomes in algal symbionts, such as increased cell size, likely due to cell cycle arrest [62] and disruption of nutrient cycling. For the latter, Rädecker et al. [63] found that heat stress destabilizes symbiotic nutrient cycling before breakdown of the coral‐alga symbiosis. Their work on *Stylophora pistillata* showed that heat stress increases the metabolic energy demand of the coral host, which is compensated by catabolic degradation of amino acids. This results in a shift from net uptake to release of ammonium by the coral holobiont, which promotes algal symbiont growth and leads to these cells retaining photosynthates, rather than providing it to the cnidarian host. This sequence of events leads to a feedback loop that decouples carbon translocation from algae to the host and contributes to symbiosis breakdown. Other work has demonstrated that thermal stress shifts the *Porites lutea* holobiont to a net heterotrophic state whereby alga‐derived nutrients do not meet host energy demands, resulting in reduced holobiont performance [64].

We could report the results of many other studies that argue for and against the OTCB, as well as other outcomes of thermal stress on algal symbionts, however each of these will be specific to the coral species studied, the heat treatment used (e.g., tank or field conditions and extent of pre‐acclimation prior to the stress regimen), the light regimen [20], and the measurement used for assessing holobiont health, be it photophysiology [65], assessment of algal pigmentation (e.g., Coral Health Chart [https://coralwatch.org/monitoring/using‐the‐chart/]), omics data, fluorescent dyes, or nutrient exchange [66]. What is clear is that corals display a wide array of responses to thermal stress—no single temperature threshold leads to oxidative stress in all coral species and their distributions, with an extreme example being the corals in the Persian Gulf, which withstand summer temperatures 10°C higher than for coral reefs in other regions [67]. Given this observation, it might prove more useful to address the basis and nature of this process at the genotype level. If, in fact, different host genotypes of the same coral species show significantly different responses to the same thermal stress treatment, then it is unlikely that a “one rule fits all” (i.e., the OTCB) exists for bleaching under normal conditions, and a more nuanced and diversified approach needs to be taken vis‐à‐vis coral restoration across the Scleractinia. This is the nascent area of multi‐omics research we address below.

## 5 What Does Gene Expression Teach Us About Coral Bleaching?

**FIGURE 1:**
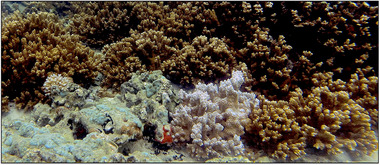
*Montipora capitata* reef in Kāne'ohe Bay, Oʻahu. This species is an obligate outbreeder and population genetic analysis of colonies in the bay demonstrates high standing genetic variation, with each colony being genetically distinct. This image shows a collection of *M. capitata* colonies in Kāne'ohe Bay of which only one has undergone bleaching (white colony) under generally the same environmental conditions. This variation in stress response is believed to be driven primarily by coral host genotype [26]. Image taken by D. Bhattacharya.

A growing body of research highlights substantial inter‐ and intra‐genotypic variation in both baseline and stress‐induced gene expression in corals [26, 68, 69]. A study published in 2018 that analyzed 30 different genotypes of *Acropora cervivornis*, from restoration nurseries in Florida exposed to heat and cold shock seasonally, identified 949 DEGs in the host that were highly variable with respect to genotype. Only a subset of four genes were consistently upregulated under heat treatment (hsp‐16.2 [heat shock] and ZFAND2B [zinc finger]) or downregulated under heat and cold treatment (ALKBH1 [alkylated DNA repair] and Drip [aquaporin]) across all genotypes [69]. Given that 100s to 1000s of genes are often found to be differentially expressed in bulk mRNA‐seq studies of corals under thermal stress [70], it may be surprising that so few are shared across different *A. cervicornis* genotypes. An analogous result, that is, highly diverged gene expression patterns among different colonies, was found in an mRNA‐seq study of *Acropora cervicornis*, highlighting the “noisy” response of these data to stress [68]. The absence of a massive, consistently shared thermal stress genetic “toolkit” (i.e., based on mRNA‐seq data) for corals has also been substantiated in other studies and suggests that the thermal stress response may vary significantly across a population (Figure 1). A review investigating over 300 papers that described omics results regarding thermal stress responses in cnidarian models found that only seven genes (CuZn‐SOD, c‐type lectin, FGFR1, MMP, Zn‐MP, NF‐κB, and SLC26) studied in at least two different genera provided consistent information about this stressor [71].

**FIGURE 2:**
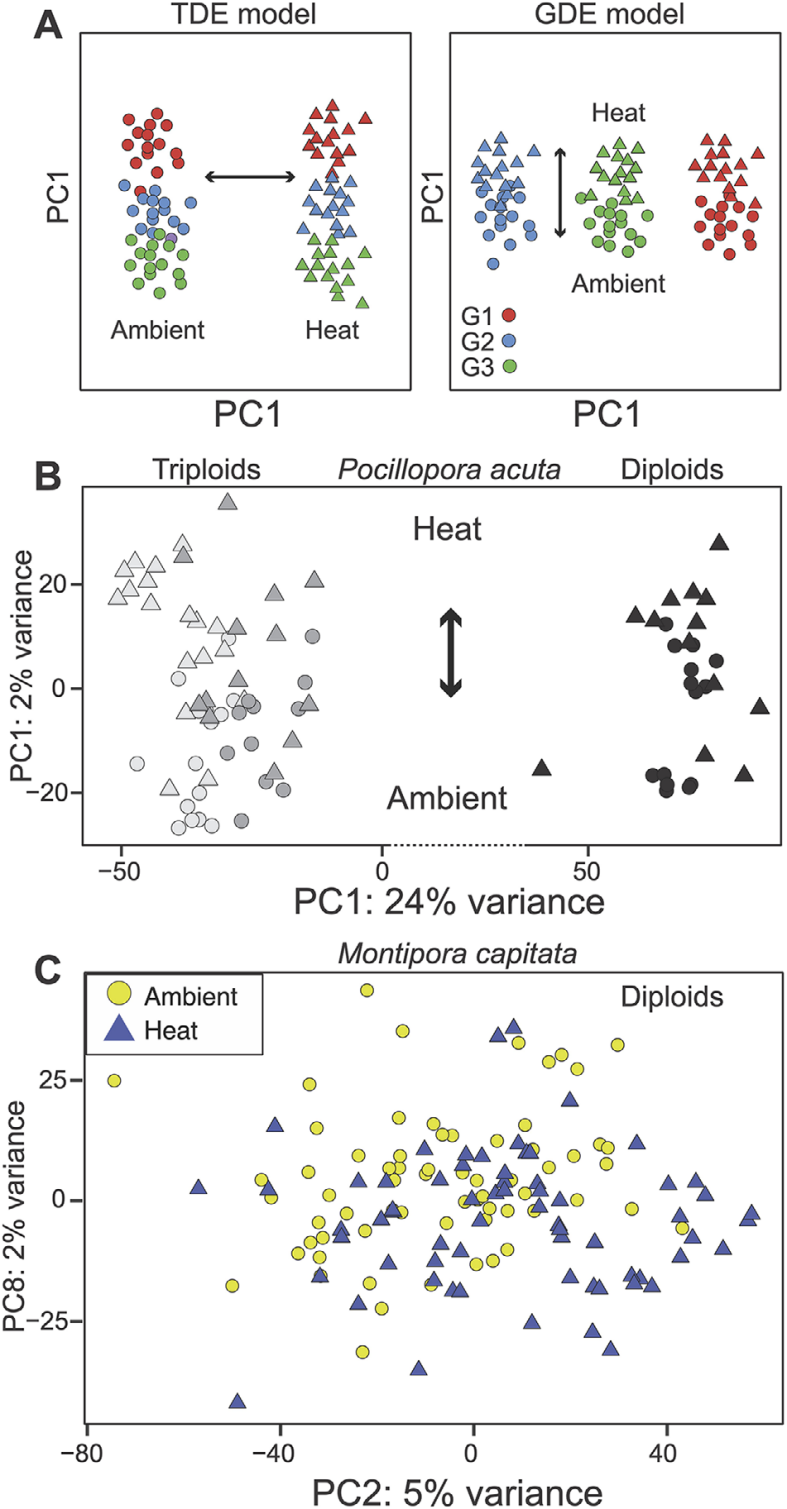
Coral animal gene expression variation under thermal stress. (A) Competing (idealized) models for the control of gene expression under thermal stress. The treatment‐driven expression (TDE) model posits that treatment is the dominant driver of the data, with different genotypes (e.g., Genotypes 1, 2, and 3; G1–G3) expressing a shared core gene set in response to thermal stress. In contrast, the genotype‐driven expression (GDE) model posits that coral genotype is the dominant driver of the stress response with the data separated by treatment within each genotype. The font size of the principal component (PC1, PC2) legends reflects the amount of expected variation (i.e., larger is greater) being explained by these components. (B) Experimental results using *Pocillopora acuta* gene expression data from the three clonal lineages, two of which are closely related triploid clades, and one a more diverged diploid clade. These data are generally consistent with the GDE model. (C) PC analysis of *Montipora capitata* gene expression data, showing that for this obligately outbreeding species, no support is found for the TDE model with each colony showing a distinct gene expression pattern, and no grouping of either ambient or heat‐treated samples. Figures (A–C) were from Chille et al. [26] under a Creative Commons Attribution 4.0 International License (http://creativecommons.org/licenses/by/4.0/). Minor modifications were made to these images.

Given the noise in a population‐level gene expression response to thermal stress, the hypothesis for genotype‐driven gene expression was tested by Chille et al. [26] using mRNA‐seq data from two Hawaiian coral species, *P. acuta* (stress sensitive) and *M. capitata* (stress resilient), that were subjected to a standard regimen of thermal and/or pH stress (see [26] for details). Key to this research is the observation that the ca. 2/3 of the *P. acuta* colonies in Kāne'ohe Bay, Oʻahu comprise three dominant, independently derived triploid clades that reproduce asexually through larval propagation or fragmentation. In contrast, the sympatric *M. capitata* in the bay is an exclusive outbreeder (i.e., via mass spawning) and virtually all colonies are genetically distinct from each other [23, 72]. This study allowed the authors to test two competing models for the regulation of gene expression in corals: treatment‐driven expression (TDE) versus genotype‐dependent expression (GDE) (Figure 2A). The former posits that treatment dominates gene expression, and under stress, different genotypes express a large, conserved gene set, whereas the latter posits that gene expression patterns primarily reflect genotype, with little overlap between different genotypes, regardless of stress. The Chille et al. data strongly support the GDE model for *P. acuta* (Figure 2B), demonstrating, and consistent with the results described above, that the shared gene expression signal among different genotypes is quite small when compared to the overall number of DEGs. In the case of *P. acuta*, even if colonies bleached or died under the stress regime, their gene expression response was distinct across genotypes (in this case, groups of colonies derived from clonal propagation and of varying ploidies [diploids and triploids]). Specifically, the low number of distinct genetic backgrounds means that the inherent differences between the clonal lineages (genotype and ploidy) dominate the transcriptome signal, effectively buffering or masking any minor, shared transcriptomic response to the heat stress. In the case of *M. capitata*, principal component analysis of the mRNA‐seq data showed no discernible pattern (Figure 2C), given that each colony is genetically diverged and therefore had a distinct response under the treatment regimens that were used [26]. These data do not support the TDE model, but the GDE model could not be tested as directly as with *P. acuta* (due to the lack of clonal replicates), that is, the overall weak signal from the treatment in *M. capitata* is attributed to the high standing genetic variation. With so many unique genotypes, the high background genetic variation made it difficult to detect a shared, population‐wide core stress response at the global transcriptome level, leading to a non‐TDE result. However, analysis of ambient and heat‐treated colonies after 1 week of exposure identified 31 DEGs out of the 18,225 genes that passed low‐abundance filtering in this study, which may represent a more limited “core” bleaching response in the population. What these data tell us is that even within a single outbreeding population, gene expression is variable, therefore it is unlikely that an overarching hypothesis or solution to coral bleaching will apply broadly across all species and populations.

The GDE model and the other data described here underscore the critical need to control and test for genotype and genotype‐by‐treatment interactions in coral gene expression studies of stress using multiple technologies (e.g., transcriptomic and proteomics) and suggest that coral bleaching is a multifactorial response that reflects many competing forces [73]. Coral animal gene expression response to thermal stress may differ significantly by genotype and other factors, such as algal symbiont population and history of heat exposure, thereby impacting bleaching outcomes. Despite a low amount of shared DEGs between genotypes, colonies may still result in convergent (bleaching) phenotypes due to post‐transcriptional processes that alter protein abundance (see below). The potential of polyploidy and hybridization as key players in coral population biology also need to be addressed in future studies of coral stress responses [23, 74]. These responses are expected to vary from species to species as well as among different genotypes of one species, making it challenging to define a clear gene expression signal predictive of the OTCB that would apply to all corals.

## 6 Are mRNA, Protein, and Metabolite Abundances Strongly Correlated Under Thermal Stress?

**FIGURE 3:**
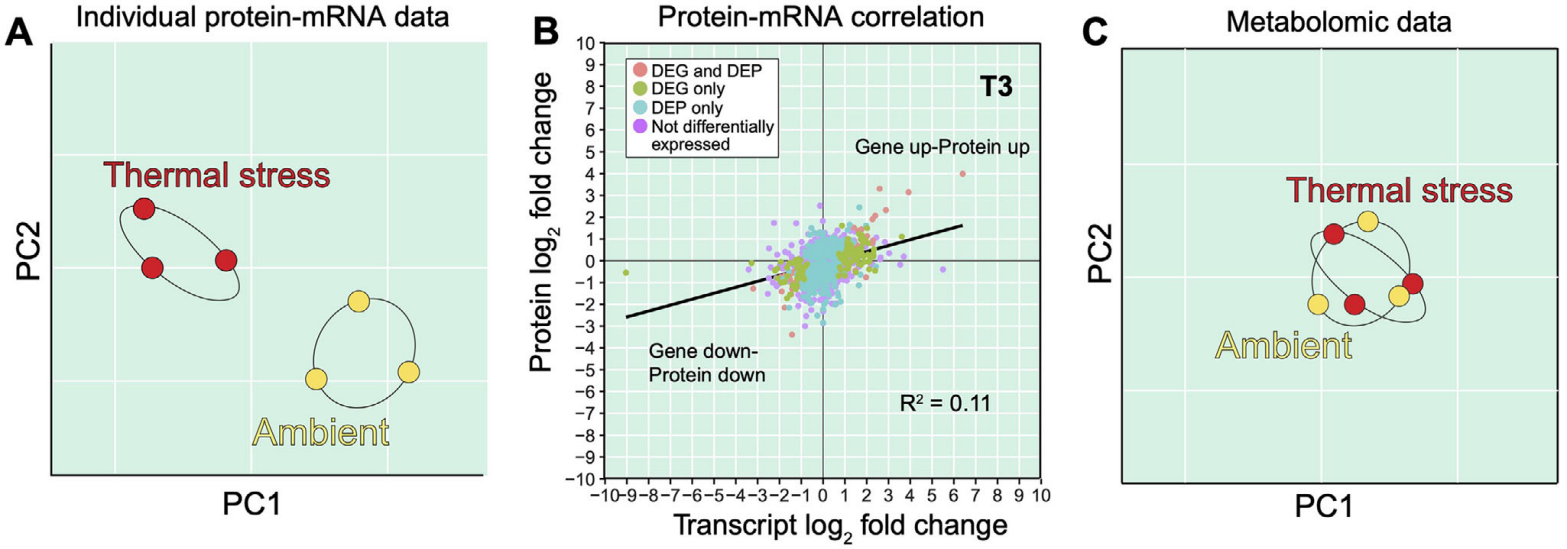
Correlation between coral multi‐omics data under thermal stress. (A) Idealized results based on multiple studies using principal component (PC) analysis that shows mRNA and protein levels individually are clearly differentiated by treatment. (B) Analysis of real data from *Montipora capitata* [76] shows however that when protein (differentially expressed proteins [DEPs]) and mRNA (differentially expressed genes [DEGs]) levels are mapped against each other, we see a very low correlation between these values. The log_2_ fold change of transcripts (*x* axis) and proteins (*y* axis) at T3 has an *R* ^2^ value of 0.11. Several genes show the “gene up‐protein up” pattern (top right quadrant) and include functions associated with the stress response (see text); however, most genes do not follow this expected trend. A trend line (black) is fitted through the data. (C) Idealized PC plot of untargeted polar metabolomic data showing the homeostatic control of metabolite levels under thermal stress that does not allow the clear separation of abundance values based on treatment. Figures (A–C) were from Williams et al. [76] under a Creative Commons Attribution 4.0 International License (http://creativecommons.org/licenses/by/4.0/). Minor modifications were made to these images.

Given the inherent noise in gene expression, do proteomics data perform better under thermal stress conditions, and are these two data sets consistent with each other (see Figure 3A)? The coral proteomics field is less well developed however some initial results are worth considering. A pioneering proteomics study of *Acropora microphthalma* from the Great Barrier Reef, Australia that exposed coral fragments to thermal stress under low and high light (full midday sunlight with average daylight PAR of 466 µmol quanta m^−2^ s^−1^) demonstrated that common host ROS‐responsive proteins (e.g., superoxide dismutase, catalase) were not present in the data under stress. This was consistent with the algal symbiont data, which showed that only a small number of antioxidants were present (not significantly more abundant) in this dataset (e.g., thiol peroxidases, xanthine dehydrogenase) [75]. A similar result was found in a more recent experiment done with *M. capitata* from Kāne'ohe Bay that had undergone short‐term thermal stress in tank cultures. Transcriptomic, proteomic, and metabolomic data were generated from the same coral fragments to allow multi‐omics data integration [76]. These results, albeit specific for the thermally resilient *M. capitata* as described above, showed very little correlation between gene expression values and the encoded protein abundance over the three timepoints used in the study (Figure 3B). In fact, only a handful of proteins showed a correlated increase in abundance under stress, including stress‐responsive proteins such as calumenin‐B (protein folding/sorting), NF‐kB (transcription factor [stress]), and mitochondrial glutamate dehydrogenase (TCA cycle). Other coral proteomic studies [77, 78] also report a more muted response to stress apparent in coral proteomes and reflect the well‐understood impacts of post‐transcriptional, translational, and protein degradation regulation [79, 80] and protein level buffering [81] that decouple these data streams. Theoretical analysis of mRNA‐protein abundance using both stabilizing and directional selection models shows a weak to no correlation between these values. This is consistent with the observation that protein levels are more evolutionarily conserved than mRNA levels, likely due to stabilizing selection acting on the former [82]. Given that protein abundance (albeit with less information content) can better predict downstream metabolite production and other functions, proteomic data are likely to be better targets for investigating the generality of the OTCB.

If the proteomic response is more muted than the transcriptomic response, then the metabolomic response is even more so. A comparative analysis of all three omics data layers in the *M. capitata* holobiont showed no clear separation (i.e., driven by a given factor) between groups apparent in the analysis of individual proteomic and transcriptomic datasets [76] (idealized result shown in Figure 3C). This suggests that a combination of stochastic (e.g., differential metabolite turnover rates) and homeostatic processes [48] act on the holobiont that are not easily untangled within the complex coral symbiosis. Specific metabolites, such as dipeptides [48], free amino acids [10], and alga‐derived betaine lipids [51] (among others), are however useful markers of coral thermal and oxidative stress and have been described elsewhere and can provide potential biomarkers of coral stress that can be developed into diagnostic tools [8]. Investigation of the holobiont metabolome vis‐à‐vis the host gene inventory using the Metabolite Annotation and Gene Integration software (MAGI) [83] demonstrated that the major response to thermal stress was the activation of animal redox stress pathways involved in quenching molecular oxygen to prevent an overabundance of ROS. This function was achieved by different cytochrome P450 family members and by upregulation of the phenylalanine‐4‐hydroxylase pathway, which uses molecular oxygen and is a response to nutrient deprivation. As described above in studies of the OTCB [60], increases in the coral metabolic rate under higher temperatures can result in physiological hyperoxia. The MAGI results suggest that the host animal provides a strong response to the production of ROS, and therefore, the enrichment of host‐derived oxidoreductases observed in the Williams et al. [48] study is an expected outcome. Metabolic data can provide insights on holobiont responses to stress however many other metabolite biomarkers of coral bleaching await to be identified once more knowledge accumulates about the significant “dark” (unidentified) metabolome present in these species [84].

How do these multi‐omics data impact our understanding of coral bleaching and the OTCB? First and foremost, the variation observed in the field and in controlled tank studies with respect to bleaching sensitivity is reflected in the multi‐omics data (and changes under different irradiance levels), which suggests the existence of vast amounts of variation that reflects the coral species, genotype, and differential acclimation to local conditions [64, 85], including composition of the algal symbiont population, and prokaryotic microbiome. Second, the host clearly plays a significant role in the response to thermal stress, and the bleaching phenotype may not primarily reflect algal symbiont production of ROS as the driver of the process. This point was underlined in a recent study of massive *Porites* species in the Great Barrier Reef. Scott et al. [86] found that the genetic structure of the host coral was the primary driver of both algal symbiont and microbiome composition. That is, the host was apparently the main arbiter of holobiont composition with the microbiome (not, however, the algal symbionts) being impacted by reef location and coral size class. Because there is less inherent noise in the proteome and metabolome, insights from these data layers can more concretely capture the complex physiological response of coral colonies to thermal stress. These data suggest that host‐derived metabolic shifts may be a primary driver of bleaching, beyond that contributed by algal symbionts under high light levels.

Finally, we briefly consider the growing amount of single‐nucleotide polymorphism (SNP) data from the coral host that promises to significantly advance understanding of coral adaptive capacity. Existing genome‐wide association studies (GWASs) show that heat tolerance is a highly polygenic trait, with hundreds to thousands of loci contributing to thermal resilience. In a study of 237 *A . millepora* from the Great Barrier Reef, Fuller et al. [87] found that no single SNP reached genome‐wide significance, suggesting that variation in bleaching is not due to common loci of large effect. Their study demonstrated that the combined, estimated effects of variants across the genome may be predictive of phenotype and that a polygenic score constructed from their results could distinguish the most bleaching‐tolerant individuals from the most susceptible, explaining >60% of total phenotypic variance when combined with other bleaching predictors such as environment and algal symbiont type. These results have direct implications for natural adaptation and potentially, conservation approaches. Given their dynamic environments, it should be selectively beneficial for corals to have complex traits controlled by many genes that respond more gradually to selection and be less constrained by lack of genetic variation. This standing genetic variation at the genome level (as described above for Hawaiian *M. capitata*) may provide a buffer against genetic bottlenecks and fine‐tunes the thermal stress response via selection on allele frequencies at multiple loci. This field of study is rapidly growing, and work in areas such as seascape genomics (genotype‐environment associations [GEAs] deduced by correlating allele frequencies with environmental gradients while accounting for the genetic structure of natural populations) [88] has identified candidate adaptive loci in genes related to stress response, cellular homeostasis, and metabolism [89].

## 7 Concluding Remarks

**FIGURE 4:**
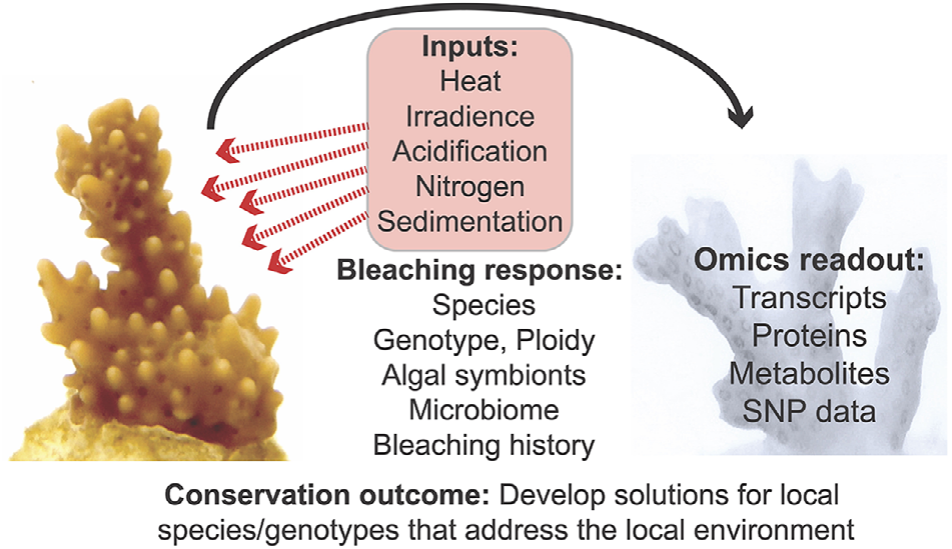
Simplified model of coral bleaching. Coral bleaching is driven by multiple factors (*Inputs*), with thermal stress and high irradiance, alone or in combination, being the most important. Other contributors such as ocean acidification and nitrogen loading may also precipitate algal symbiont expulsion. The *Bleaching response* to abiotic stress is multifactorial with host animal species/strain and algal symbiont genotypes being the most critical. Other factors such as past thermal stress events can lead to “frontloading” [54] that provides colony resilience to thermal stress. The *Omics readout* to thermal stress is complex with gene expression being most “noisy” and impacted primarily by genotype and local adaptation. Proteome and metabolome data show less variation but are more informative about coral stress state. The interaction of all these variables makes it very challenging to define coral bleaching under a single over‐arching hypothesis such as the Oxidative Theory of Coral Bleaching (OTCB), although oxidative stress (animal, algal symbiont, or both) clearly plays a role in the bleaching phenotype. These studies suggest that *Conservation outcomes* may best be addressed with a personal genomics approach, whereby local reefs are characterized with respect to genotype and other contributors to bleaching resilience to develop local solutions.

The OTCB provides an explanatory framework for understanding coral bleaching, particularly in the context of biochemical and cellular responses to environmental stress (Figure 4). More importantly, the OTCB provides a model for more broadly understanding how coral holobionts respond to fluctuating environmental conditions. The strength of this theory lies in providing a mechanistic understanding of how environmental stress translates into physiological damage. However, a major difficulty lies in establishing causality between ROS production and oxidative damage that are commonly observed during bleaching. It is possible, and we argue more likely, that bleaching is a consequence of holobiont stress rather than being explained solely by algal photosynthetic impairment. However, unmitigated oxidative stress will clearly have serious repercussions for coral health (or that of any other organism). We presume that under most tolerable conditions, ROS acts as a signaling molecule [90] rather than as a direct cytotoxic agent that drives algal expulsion. We suggest that any explanatory framework for understanding coral bleaching should be integrated within a broader ecological, physiological, and genetic context to explain the differences in the resistance and resilience between different species, populations, and genotypes. The use of multi‐omics, SNP, and other genome‐tdewide data to address this central issue has proven valuable, not by simplifying the problem vis‐à‐vis identifying a small set of variables or genotypes that faithfully predict bleaching across all corals (as hoped). Rather, multi‐omics and GWAS data have revealed the vast diversity of thermal stress responses that have evolved in Scleractinia over their geographic ranges. These data therefore offer the foundation for rational planning of coral conservation efforts that are species/genotype and location specific. This approach mirrors closely that taken to human health in the expanding field of personal genomics [91].

## Author Contributions

**Debashish Bhattacharya**: conceptualization, visualization, writing – original draft. **Shrinivas Nandi**: writing – review and editing. **Erin E. Chille**: writing – review and editing. **Miriam Arroyo**: writing – review and editing. **Timothy G. Stephens**: writing – review and editing. All authors reviewed and approved the final version of the manuscript.

## Conflicts of Interest

The authors declare no conflicts of interest.

## Data Availability

The datasets that support the findings of this study are publicly available. No unique datasets were generated for this review article.

## References

[1] J. E. N. Veron , “Scleractinia, Evolution and Taxonomy,” in Encyclopedia of Modern Coral Reefs: Structure, Form and Process (Springer, 2011), 947–957.

[2] S. W. Davies , M. H. Gamache , L. I. Howe‐Kerr , et al., “Building Consensus Around the Assessment and Interpretation of Symbiodiniaceae Diversity,” PeerJ 11 (2023): 15023, 10.7717/peerj.15023. PMC10162043 37151292

[3] T. W. Davies , O. Levy , S. Tidau , et al., “Global Disruption of Coral Broadcast Spawning Associated With Artificial Light at Night,” Nature Communications 14 (2023): 2511, 10.1038/s41467-023-38070-y. PMC10185496 37188683

[4] D. Bhattacharya , T. G. Stephens , E. E. Chille , L. F. Benites , and C. X. Chan , “Facultative Lifestyle Drives Diversity of Coral Algal Symbionts,” Trends in Ecology & Evolution 39 (2024): 239–247, 10.1016/j.tree.2023.10.005. 37953106

[5] F. Wiederkehr , K. E. Engelhardt , J. Vetter , et al., “Host‐Level Biodiversity Shapes the Dynamics and Networks Within the Coral Reef Microbiome,” ISME Communications 5, no. 1 (2025): ycaf097, 10.1093/ismeco/ycaf097. 40568303 PMC12192423

[6] V. M. Glynn , L. F. de Barros Marangoni , M. Guglielmetti , et al., “The Role of Holobiont Composition and Environmental History in Thermotolerance of Tropical Eastern Pacific Corals,” Current Biology 35 (2025): 3048–3063.e7, 10.1016/j.cub.2025.05.035. 40480235

[7] A. S. Huffmyer , K. H. Wong , D. M. Becker , E. Strand , T. Mass , and H. M. Putnam , “Shifts and Critical Periods in Coral Metabolism Reveal Energetic Vulnerability During Development,” Current Biology 35 (2025): 2858–2871.e6, 10.1016/j.cub.2025.05.013. 40441134

[8] E. E. Chille , T. G. Stephens , S. Nandi , et al., “Coral Restoration in the Omics Era: Development of Point‐of‐Care Tools for Monitoring Disease, Reproduction, and Thermal Stress,” BioEssays 47 (2025): 70007, 10.1002/bies.70007. PMC12101048 40285547

[9] M. J. H. van Oppen and M. Medina , “Coral Evolutionary Responses to Microbial Symbioses,” Philosophical transactions of the Royal Society of London Series B, Biological Sciences 375 (2020): 20190591, 10.1098/rstb.2019.0591. 32772672 PMC7435167

[10] N. Rädecker , S. Escrig , J. E. Spangenberg , C. R. Voolstra , and A. Meibom , “Coupled Carbon and Nitrogen Cycling Regulates the Cnidarian–Algal Symbiosis,” Nature Communications 14 (2023): 6948, 10.1038/s41467-023-42579-7. PMC10620199 37914705

[11] O. Hoegh‐Guldberg , “Climate Change, Coral Bleaching and the Future of the World's Coral Reefs,” Marine & Freshwater Research 50 (1999): 839–866, 10.1071/MF99078.

[12] R. Iglesias‐Prieto and R. K. Trench , “Acclimation and Adaptation to Irradiance in Symbiotic Dinoflagellates. I. Responses of the Photosynthetic Unit to Changes in Photon Flux Density,” Marine Ecology Progress Series 113 (1994): 163–175, 10.3354/meps113163.

[13] A. C. Baker , P. W. Glynn , and B. Riegl , “Climate Change and Coral Reef Bleaching: An Ecological Assessment of Long‐Term Impacts, Recovery Trends and Future Outlook,” Estuarine, Coastal and Shelf Science 80 (2008): 435–471, 10.1016/j.ecss.2008.09.003.

[14] D. Burn , M. Álvarez‐Noriega , and R. Ferrari , “Keystone Coral Species Population Collapse After Unprecedented Heat Stress,” Trends in Ecology & Evolution 40 (2025): 1164–1166, 10.1016/j.tree.2025.10.006. 41198432

[15] B. E. Brown , “Coral Bleaching: Causes and Consequences,” Coral Reefs 16 (1997): 129–138, 10.1007/s003380050249.

[16] J. Helgoe , S. K. Davy , V. M. Weis , and M. Rodriguez‐Lanetty , “Triggers, Cascades, and Endpoints: Connecting the Dots of Coral Bleaching Mechanisms,” Biological Reviews of the Cambridge Philosophical Society 99 (2024): 715–752, 10.1111/brv.13042. 38217089

[17] S. G. Klein , N. R. Geraldi , A. Anton , et al., “Projecting Coral Responses to Intensifying Marine Heatwaves Under Ocean Acidification,” Global Change Biology 28 (2022): 1753–1765, 10.1111/gcb.15818. 34343392 PMC9291544

[18] M. P. Lesser , W. R. Stochaj , D. W. Tapley , and J. M. Shick , “Bleaching in Coral Reef Anthozoans: Effects of Irradiance, Ultraviolet Radiation, and Temperature on the Activities of Protective Enzymes Against Active Oxygen,” Coral Reefs 8 (1990): 225–232, 10.1007/BF00265015.

[19] C. A. Downs , J. E. Fauth , J. C. Halas , P. Dustan , J. Bemiss , and C. M. Woodley , “Oxidative Stress and Seasonal Coral Bleaching,” Free Radical Biology & Medicine 33 (2002): 533–543, 10.1016/S0891-5849(02)00907-3. 12160935

[20] M. P. Lesser , “Irradiance Dependency of Oxidative Stress and Coral Bleaching,” Coral Reefs 43 (2024): 1393–1403, 10.1007/s00338-024-02545-1.

[21] J. M. Pandolfi , R. H. Bradbury , E. Sala , et al., “Global Trajectories of the Long‐Term Decline of Coral Reef Ecosystems,” Science 301 (2003): 955–958, 10.1126/science.1085706. 12920296

[22] T. Krueger , T. D. Hawkins , S. Becker , et al., “Differential Coral Bleaching—Contrasting the Activity and Response of Enzymatic Antioxidants in Symbiotic Partners Under Thermal Stress,” Comparative Biochemistry and Physiology Part A, Molecular & Integrative Physiology 190 (2015): 15–25, 10.1016/j.cbpa.2015.08.012. 26310104

[23] T. G. Stephens , E. L. Strand , H. M. Putnam , and D. Bhattacharya , “Ploidy Variation and Its Implications for Reproduction and Population Dynamics in Two Sympatric Hawaiian Coral Species,” Genome Biology and Evolution 15 (2023): evad149, 10.1093/gbe/evad149. 37566739 PMC10445776

[24] C. Drury , D. Manzello , and D. Lirman , “Genotype and Local Environment Dynamically Influence Growth, Disturbance Response and Survivorship in the Threatened Coral, *Acropora cervicornis* ,” PLoS ONE 12 (2017): 0174000, 10.1371/journal.pone.0174000. PMC5358778 28319134

[25] C. Drury and D. Lirman , “Genotype by Environment Interactions in Coral Bleaching,” Proceedings Biological Sciences 288 (2021): 20210177. 33653132 10.1098/rspb.2021.0177 PMC7934956

[26] E. E. Chille , T. G. Stephens , D. Misri , E. L. Strand , H. M. Putnam , and D. Bhattacharya , “Gene Expression Response Under Thermal Stress in Two Hawaiian Corals Is Dominated by Ploidy and Genotype,” Ecology and Evolution 14 (2024): 70037, 10.1002/ece3.70037. PMC11268936 39050655

[27] K. E. Dougan , A. J. Bellantuono , T. Kahlke , et al., “Whole‐Genome Duplication in an Algal Symbiont Bolsters Coral Heat Tolerance,” Science Advances 10 (2024): adn2218, 10.1126/sciadv.adn2218. PMC11259175 39028812

[28] G. D. Stanley Jr , “The Evolution of Modern Corals and Their Early History,” Earth‐Science Reviews 60 (2003): 195–225, 10.1016/S0012-8252(02)00104-6.

[29] C. D. Kenkel and L. K. Bay , “Exploring Mechanisms That Affect Coral Cooperation: Symbiont Transmission Mode, Cell Density and Community Composition,” PeerJ 6 (2018): 6047, 10.7717/peerj.6047. PMC6282938 30533318

[30] C. M. Dunphy , S. V. Vollmer , and T. C. Gouhier , “Host–Microbial Systems as Glass Cannons: Explaining Microbiome Stability in Corals Exposed to Extrinsic Perturbations,” Journal of Animal Ecology 90 (2021): 1044–1057, 10.1111/1365-2656.13466. 33666231

[31] M. Ziegler , C. G. B. Grupstra , M. M. Barreto , et al., “Coral Bacterial Community Structure Responds to Environmental Change in a Host‐Specific Manner,” Nature Communications 10 (2019): 3092, 10.1038/s41467-019-10969-5. PMC6626051 31300639

[32] J. M. Casey , S. R. Connolly , and T. D. Ainsworth , “Coral Transplantation Triggers Shift in Microbiome and Promotion of Coral Disease Associated Potential Pathogens,” Scientific Reports 5 (2015): 11903, 10.1038/srep11903. 26144865 PMC4491727

[33] B. A. Wallace , N. S. Varona , A. K. Stiffler , M. J. A. Vermeij , and C. Silveira , “High Microbial Diversity, Functional Redundancy, and Prophage Enrichment Support the Success of the Yellow Pencil Coral, *Madracis mirabilis*, in Curaçao's Coral Reefs,” mSystems 10 (2025): 0120825, 10.1128/msystems.01208-25. PMC12625765 41099510

[34] N. Garcias‐Bonet , H. Villela , F. C. García , et al., “The Coral Probiotics Village: An Underwater Laboratory to Tackle the Coral Reefs Crisis,” Ecology and Evolution 15 (2025): 71558, 10.1002/ece3.71558. PMC12231234 40625325

[35] M. P. Lesser , “Oxidative Stress in Marine Environments: Biochemistry and Physiological Ecology,” Annual Review of Physiology 68 (2006): 253–278, 10.1146/annurev.physiol.68.040104.110001. 16460273

[36] I. Zilber‐Rosenberg and E. Rosenberg , “Role of Microorganisms in the Evolution of Animals and Plants: The Hologenome Theory of Evolution,” FEMS Microbiology Reviews 32 (2008): 723–735, 10.1111/j.1574-6976.2008.00123.x. 18549407

[37] J. Suárez and A. Stencel , “A Part‐Dependent Account of Biological Individuality: Why Holobionts Are Individuals and Ecosystems Simultaneously,” Biological Reviews of the Cambridge Philosophical Society 95 (2020): 1308–1324, 10.1111/brv.12610. 32406121

[38] E. A. Lloyd and M. J. Wade , “Criteria for Holobionts From Community Genetics,” Biological Theory 14 (2019): 151–170, 10.1007/s13752-019-00322-w.

[39] E. A. Tripp , N. Zhang , H. Schneider , et al., “Reshaping Darwin's Tree: Impact of the Symbiome,” Trends in Ecology & Evolution 32 (2017): 552–555, 10.1016/j.tree.2017.05.002. 28601483

[40] S. M. Rosales , L. K. Huebner , A. S. Clark , R. McMinds , R. R. Ruzicka , and E. M. Muller , “Bacterial Metabolic Potential and Micro‐Eukaryotes Enriched in Stony Coral Tissue Loss Disease Lesions,” Frontiers in Marine Science 8 (2022): 776859, 10.3389/fmars.2021.776859.

[41] T. M. Work , T. M. Weatherby , J. H. Landsberg , Y. Kiryu , S. M. Cook , and E. C. Peters , “Viral‐Like Particles Are Associated with Endosymbiont Pathology in Florida Corals Affected by Stony Coral Tissue Loss Disease,” Frontiers in Marine Science 8 (2021): 750658, 10.3389/fmars.2021.750658.

[42] S. Nandi , T. G. Stephens , K. Walsh , et al., “Shifts in the Microbiome and Virome Are Associated With Stony Coral Tissue Loss Disease (SCTLD),” ISME Communications 5 (2025): ycaf226, 10.1093/ismeco/ycaf226. 41459349 PMC12743298

[43] A. J. Bellantuono , K. E. Dougan , C. Granados‐Cifuentes , and M. Rodriguez‐Lanetty , “Free‐Living and Symbiotic Lifestyles of a Thermotolerant Coral Endosymbiont Display Profoundly Distinct Transcriptomes Under Both Stable and Heat Stress Conditions,” Molecular Ecology 28 (2019): 5265–5281, 10.1111/mec.15300. 31693775

[44] S. J. Robbins , C. M. Singleton , C. X. Chan , et al., “A Genomic View of the Reef‐Building Coral *Porites lutea* and Its Microbial Symbionts,” Nature Microbiology 4 (2019): 2090–2100, 10.1038/s41564-019-0532-4. 31548681

[45] Y. J. Liew , Y. Li , S. Baumgarten , C. R. Voolstra , and M. Aranda , “Condition‐Specific RNA Editing in the Coral Symbiont *Symbiodinium microadriaticum* ,” PLoS Genetics 13 (2017): 1006619, 10.1371/journal.pgen.1006619. PMC5357065 28245292

[46] A. Moustafa , A. N. Evans , D. M. Kulis , et al., “Transcriptome Profiling of a Toxic Dinoflagellate Reveals a Gene‐Rich Protist and a Potential Impact on Gene Expression Due to Bacterial Presence,” PLoS ONE 5 (2010): 9688, 10.1371/journal.pone.0009688. PMC2837391 20300646

[47] S. Nandi , T. G. Stephens , E. E. Chille , S. Goyen , L. K. Bay , and D. Bhattacharya , “Metaproteome Analysis of Short‐Term Thermal Stress in Three Sympatric Coral Species Reveals Divergent Host Responses,” bioRxiv (2025), 10.1101/2025.03.25.645042. PMC13093290 42016983

[48] A. Williams , E. N. Chiles , D. Conetta , et al., “Metabolomic Shifts Associated With Heat Stress in Coral Holobionts,” Science Advances 7 (2021): abd4210, 10.1126/sciadv.abd4210. PMC7775768 33523848

[49] J. R. Garcia and N. M. Gerardo , “The Symbiont Side of Symbiosis: Do Microbes Really Benefit?” Frontiers in Microbiology 5 (2014): 510, 10.3389/fmicb.2014.00510. 25309530 PMC4176458

[50] A. A. Mushegian and D. Ebert , “Rethinking “Mutualism” in Diverse Host‐Symbiont Communities,” BioEssays 38 (2016): 100–108, 10.1002/bies.201500074. 26568407

[51] G. Tortorelli , S. L. Rosset , C. E. S. Sullivan , et al., “Heat‐Induced Stress Modulates Cell Surface Glycans and Membrane Lipids of Coral Symbionts,” The ISME Journal 19 (2025): wraf073, 10.1093/ismejo/wraf073. 40247696 PMC12077390

[52] A. M. Dungan , D. Bulach , H. Lin , M. J. H. van Oppen , and L. L. Blackall , “Development of a Free Radical Scavenging Bacterial Consortium to Mitigate Oxidative Stress in Cnidarians,” Microbial Biotechnology 14 (2021): 2025–2040, 10.1111/1751-7915.13877. 34259383 PMC8449677

[53] E. L. Strand , K. H. Wong , A. Farraj , S. Gray , A. McMenamin , and H. M. Putnam , “Coral Species‐Specific Loss and Physiological Legacy Effects Are Elicited by an Extended Marine Heatwave,” Journal of Experimental Biology 227 (2024): jeb246812, 10.1242/jeb.246812. 38774956

[54] D. J. Barshis , J. T. Ladner , T. A. Oliver , F. O. Seneca , N. Traylor‐Knowles , and S. R. Palumbi , “Genomic Basis for Coral Resilience to Climate Change,” Proceedings of the National Academy of Sciences of the United States of America 110 (2013): 1387–1392, 10.1073/pnas.1210224110. 23297204 PMC3557039

[55] D. J. Suggett , M. E. Warner , D. J. Smith , P. Davey , S. Hennige , and N. R. Baker , “Photosynthesis and Production Of Hydrogen Peroxide by Symbiodinium (Pyrrhophyta) Phylotypes With Different Thermal Tolerances 1,” Journal of Phycology 44 (2008): 948–956, 10.1111/j.1529-8817.2008.00537.x. 27041613

[56] M. Rezayian , V. Niknam , and H. Ebrahimzadeh , “Oxidative Damage and Antioxidative System in Algae,” Toxicology Reports 6 (2019): 1309–1313, 10.1016/j.toxrep.2019.10.001. 31993331 PMC6978204

[57] Y. Fichman , L. Rowland , M. J. Oliver , and R. Mittler , “ROS Are Evolutionary Conserved Cell‐to‐Cell Stress Signals,” Proceedings of the National Academy of Sciences of the United States of America 120 (2023): 2305496120, 10.1073/pnas.2305496120. PMC10400990 37494396

[58] M. E. Warner , W. K. Fitt , and G. W. Schmidt , “Damage to Photosystem II in Symbiotic Dinoflagellates: A Determinant of Coral Bleaching,” Proceedings of the National Academy of Sciences of the United States of America 96 (1999): 8007–8012, 10.1073/pnas.96.14.8007. 10393938 PMC22178

[59] D. A. Nielsen , K. Petrou , and R. D. Gates , “Coral Bleaching From a Single Cell Perspective,” The ISME Journal 12 (2018): 1558–1567, 10.1038/s41396-018-0080-6. 29463894 PMC5955907

[60] S. R. Dunn , M. Pernice , K. Green , et al., “Regulation of Apoptosis by Mitochondrial Reactive Oxygen Species in the Coral *Acropora millepora* ,” PLoS ONE 7 (2012): 37217.

[61] M. Schlotheuber , C. R. Voolstra , C. de Beer , et al., “High Temporal Resolution of Hydrogen Peroxide (H_2_O_2_) Dynamics During Heat Stress Does Not Support a Causative Role in Coral Bleaching,” Coral Reefs 43 (2024): 119–133, 10.1007/s00338-023-02448-7.

[62] M. Amario , L. B. Villela , D. Jardim‐Messeder , et al., “Physiological Response of Symbiodiniaceae to Thermal Stress: Reactive Oxygen Species, Photosynthesis, and Relative Cell Size,” PLoS ONE 18 (2023): 0284717, 10.1371/journal.pone.0284717. PMC10399794 37535627

[63] N. Rädecker , C. Pogoreutz , H. M. Gegner , et al., “Heat Stress Destabilizes Symbiotic Nutrient Cycling in Corals,” Proceedings of the National Academy of Sciences of the United States of America 118 (2021): 2022653118, 10.1073/pnas.2022653118. PMC7865147 33500354

[64] E. Marangon , N. Rädecker , J. Y. Q. Li , et al., “Destabilization of Mutualistic Interactions Shapes the Early Heat Stress Response of the Coral Holobiont,” Microbiome 13 (2025): 31, 10.1186/s40168-024-02006-5. 39891167 PMC11783734

[65] S. L. Ellis , M. E. Baird , L. P. Harrison , K. G. Schulz , and D. P. Harrison , “A Photophysiological Model of Coral Bleaching Under Light and Temperature Stress: Experimental Assessment,” Conservation Physiology 13 (2025): coaf020, 10.1093/conphys/coaf020. 40235654 PMC11997550

[66] F. Pfab , A. R. Detmer , H. V. Moeller , R. M. Nisbet , H. M. Putnam , and R. Cunning , “Heat Stress and Bleaching in Corals: A Bioenergetic Model,” Coral Reefs 43 (2024): 1627–1645, 10.1007/s00338-024-02561-1. 39553893 PMC11561010

[67] S. L. Coles and B. M. Riegl , “Thermal Tolerances of Reef Corals in the Gulf: A Review of the Potential for Increasing Coral Survival and Adaptation to Climate Change Through Assisted Translocation,” Marine Pollution Bulletin 72 (2013): 323–332, 10.1016/j.marpolbul.2012.09.006. 23058810

[68] J. Dilworth , W. C. Million , M. Ruggeri , et al., “Synergistic Response to Climate Stressors in Coral Is Associated With Genotypic Variation in Baseline Expression,” Proceedings Biological Sciences 291 (2024): 20232447. 38531406 10.1098/rspb.2023.2447 PMC10965326

[69] J. E. Parkinson , E. Bartels , M. K. Devlin‐Durante , et al., “Extensive Transcriptional Variation Poses a Challenge to Thermal Stress Biomarker Development for Endangered Corals,” Molecular Ecology 27 (2018): 1103–1119, 10.1111/mec.14517. 29412490

[70] A. Shumaker , H. M. Putnam , H. Qiu , et al., “Genome Analysis of the Rice Coral *Montipora capitata* ,” Scientific Reports 9 (2019): 2571, 10.1038/s41598-019-39274-3. 30796282 PMC6385260

[71] C. G. Molinari , C. McDougall , and K. A. Pitt , “Understanding Dynamic Molecular Responses Is Key to Designing Environmental Stress Experiments: A Review of Gene and Protein Expression in Cnidaria Under Stress,” Molecular Ecology 34 (2025): 17753, 10.1111/mec.17753. PMC12010465 40170371

[72] C. Caruso , M. Rocha de Souza , L. Ruiz‐Jones , et al., “Genetic Patterns in *Montipora capitata* Across an Environmental Mosaic in Kāne'ohe Bay, O'ahu, Hawai'i,” Molecular Ecology 31 (2022): 5201–5213, 10.1111/mec.16655. 35962751 PMC9825948

[73] V. M. Glynn , L. F. de Barros Marangoni , M. Guglielmetti , et al., “The Role of Holobiont Composition and Environmental History in Thermotolerance of Tropical Eastern Pacific Corals,” Current Biology 35 (2025): 3048–3063.e7, 10.1016/j.cub.2025.05.035. 40480235

[74] M. Furukawa , A. I. Tarigan , S. Kitanobo , N. Hanahara , S. Ohki , and M. Morita , “Introgression and Adaptive Potential Following Heavy Bleaching Events in *Acropora* Corals,” Current Biology 35 (2025): 3064–3075, 10.1016/j.cub.2025.05.038. 40494361

[75] A. J. Weston , W. C. Dunlap , V. H. Beltran , et al., “Proteomics Links the Redox State to Calcium Signaling During Bleaching of the Scleractinian Coral *Acropora microphthalma* on Exposure to High Solar Irradiance and Thermal Stress,” Molecular & Cellular Proteomics 14 (2015): 585–595, 10.1074/mcp.M114.043125. 25561505 PMC4349979

[76] A. Williams , T. G. Stephens , A. Shumaker , and D. Bhattacharya , “Peeling Back the Layers of Coral Holobiont Multi‐Omics Data,” iScience 26 (2023): 107623, 10.1016/j.isci.2023.107623. 37694134 PMC10482995

[77] M. Ricaurte , N. V. Schizas , P. Ciborowski , and N. M. Boukli , “Proteomic Analysis of Bleached and Unbleached *Acropora palmata* , a Threatened Coral Species of the Caribbean,” Marine Pollution Bulletin 107 (2016): 224–232, 10.1016/j.marpolbul.2016.03.068. 27105725 PMC6540970

[78] H. Ma , W. Dellisanti , J. T. Hao Chung , et al., “Proteomic Insights Into the Environmental Adaptation of the Subtropical Brain Coral Host *Platygyra carnosa* ,” iScience 28 (2025): 112287, 10.1016/j.isci.2025.112287. 40248114 PMC12005889

[79] L. Ponnala , Y. Wang , Q. Sun , and K. J. van Wijk , “Correlation of mRNA and Protein Abundance in the Developing Maize Leaf,” The Plant Journal 78 (2014): 424–440, 10.1111/tpj.12482. 24547885

[80] D. Jiang , A. L. Cope , J. Zhang , and M. Pennell , “On the Decoupling of Evolutionary Changes in mRNA and Protein Levels,” Molecular Biology and Evolution 40 (2023): msad169, 10.1093/molbev/msad169. 37498582 PMC10411491

[81] Y. Liu , A. Beyer , and R. Aebersold , “On the Dependency of Cellular Protein Levels on mRNA Abundance,” Cell 165 (2016): 535–550, 10.1016/j.cell.2016.03.014. 27104977

[82] D. F. Wilson and F. M. Matschinsky , “Metabolic Homeostasis in Life as We Know It: Its Origin and Thermodynamic Basis,” Frontiers in Physiology 12 (2021): 658997, 10.3389/fphys.2021.658997. 33967829 PMC8104125

[83] O. Erbilgin , O. Rübel , K. B. Louie , et al., “MAGI: A Method for Metabolite Annotation and Gene Integration,” ACS Chemical Biology 14 (2019): 704–714, 10.1021/acschembio.8b01107. 30896917

[84] R. R. da Silva , P. C. Dorrestein , and R. A. Quinn , “Illuminating the Dark Matter in Metabolomics,” Proceedings of the National Academy of Sciences of the United States of America 112 (2015): 12549–12550, 10.1073/pnas.1516878112. 26430243 PMC4611607

[85] M. Dias , A. Ferreira , R. Gouveia , et al., “Long‐Term Exposure to Increasing Temperatures on Scleractinian Coral Fragments Reveals Oxidative Stress,” Marine Environmental Research 150 (2019): 104758, 10.1016/j.marenvres.2019.104758. 31301459

[86] C. B. Scott , R. Schott , and M. V. Matz , “Genetic Clustering Within Massive *Porites* Species Complex Is the Primary Driver of Holobiont Assembly,” PLoS ONE 20 (2025): 0328479, 10.1371/journal.pone.0328479. PMC12270097 40674308

[87] Z. L. Fuller , V. J. L. Mocellin , L. A. Morris , et al., “Population Genetics of the Coral *Acropora millepora*: Toward Genomic Prediction of Bleaching,” Science 369 (2020): aba4674, 10.1126/science.aba4674. 32675347

[88] L. Liggins , E. A. Treml , and C. Riginos , “Seascape Genomics: Contextualizing Adaptive and Neutral Genomic Variation in the Ocean environment,” in Population Genomics: Marine Organisms (Springer, 2019), 171–217.

[89] O. Selmoni , G. Lecellier , H. Magalon , et al., “Seascape Genomics Reveals Candidate Molecular Targets of Heat Stress Adaptation in Three Coral Species,” Molecular Ecology 30 (2021): 1892–1906, 10.1111/mec.15857. 33619812 PMC8252710

[90] M. Schieber and N. S. Chandel , “ROS Function in Redox Signaling and Oxidative Stress,” Current Biology 24 (2014): R453–R462, 10.1016/j.cub.2014.03.034. 24845678 PMC4055301

[91] H. L. Rehm , “Evolving Health Care Through Personal Genomics,” Nature Reviews Genetics 18 (2017): 259–267, 10.1038/nrg.2016.162. PMC6517837 28138143

